# Supplementary material for: Transcriptome Analysis of In Vitro Fertilization and Parthenogenesis Activation during Early Embryonic Development in Pigs
Source: Genes (Basel). 2021 Sep 22;12(10):1461. doi: 10.3390/genes12101461 (PMC8535918; doi:10.3390/genes12101461)
Supplement: Supplementary file 1 [file genes-12-01461-s001.zip › supplementary fig S2 gene distribution.pdf]

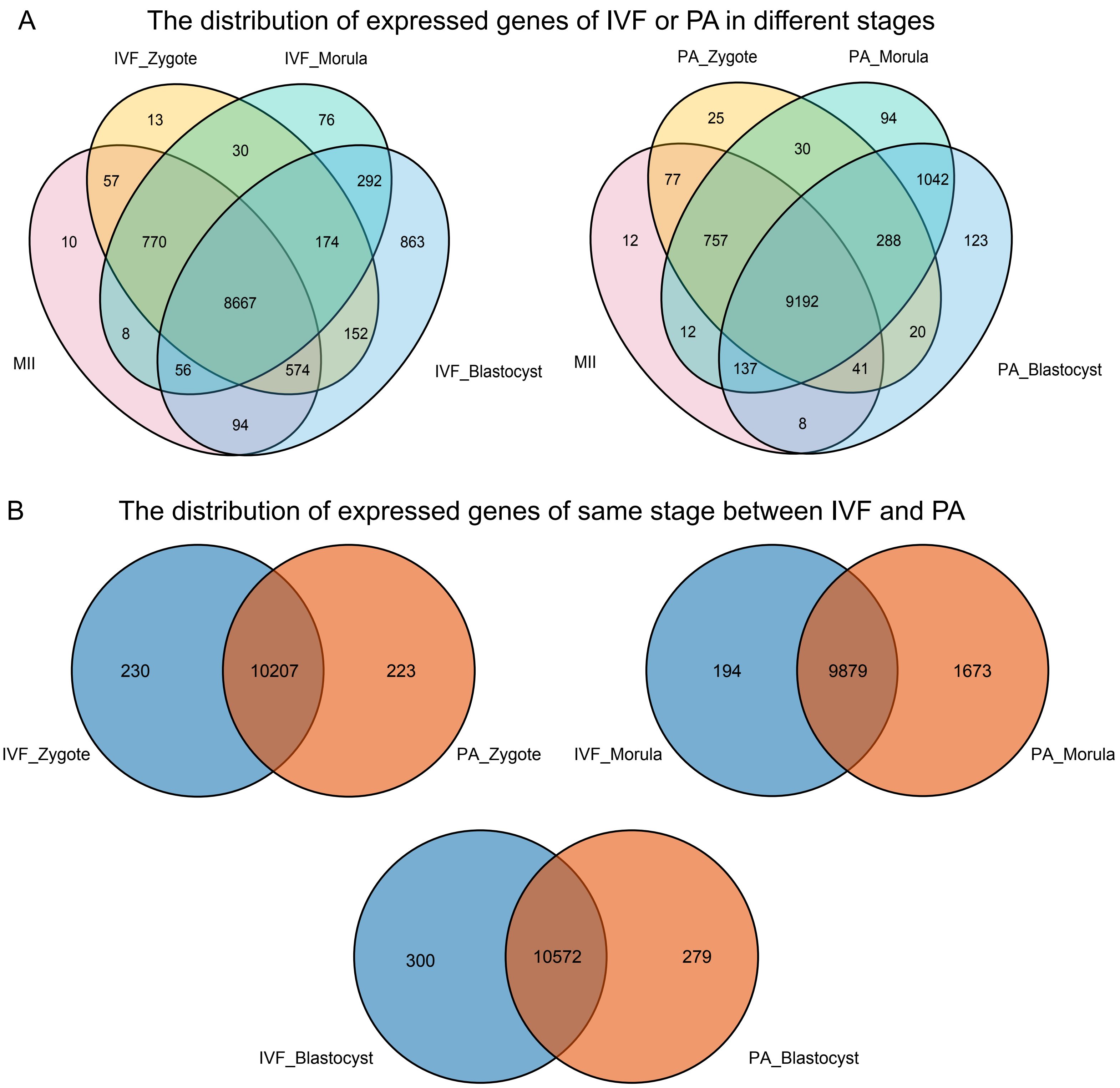
A
The distribution of expressed genes of IVF or PA in different stages
IVF_Zygote
IVF_Morula
MII
IVF_Blastocyst
13
76
30
57
292
10
770
174
863
8667
8
152
56
574
94
PA_Zygote
PA_Morula
MII
PA_Blastocyst
25
94
30
77
1042
12
757
288
123
9192
12
20
137
41
8
B
The distribution of expressed genes of same stage between IVF and PA
230
10207
223
IVF_Zygote
PA_Zygote
194
9879
1673
IVF_Morula
PA_Morula
300
10572
279
IVF_Blastocyst
PA_Blastocyst
