# Supplementary material for: Transcriptome Analysis of In Vitro Fertilization and Parthenogenesis Activation during Early Embryonic Development in Pigs
Source: Genes (Basel). 2021 Sep 22;12(10):1461. doi: 10.3390/genes12101461 (PMC8535918; doi:10.3390/genes12101461)
Supplement: Supplementary file 1 [file genes-12-01461-s001.zip › supplementary figure table legends.pdf]

Figure Legends

Table 1 In vitro embryo development of IVM oocytes after IVF or PA

Table 2 Summary of data from RNA-seq in MII, IVF and PA

Figure 1 Unsupervised clustering of the expression profiles and DEG between adjacent stages of pig embryos in vitro fertilization (IVF).

(A): Unsupervised hierarchical clustering of the expression profiles; (B): Clusters of DEGs between adjacent stages of pig embryos in vitro fertilization (IVF). Gene average log transformed expression values, top GO terms, and corresponding enrichment P-values were listed.

Figure 2 Clusters of DEGs between two adjacent stages of pig parthenogenesis activation (PA) embryos. Gene average log transformed expression values, top GO terms, and corresponding enrichment P-values were listed.

Figure 3 Top Go terms of its neighboring protein-coding genes

Figure 4 Module-stage correlation analysis and co-expression network analysis of differentially expressed novel lincRNAs and protein-coding genes.

(A): Module-stage correlations and corresponding P values, On the left, different colors represent different modules; on the right, red indicates positive correlation, white indicates none correlation, blue indicates negative correlation; each cell contains the correlation and P value given in parentheses. (B): Co-expression networks of differentially expressed novel lincRNAs and protein-coding genes in 6 modules, Top of each panel: heat maps for expression level of co-expressed genes in six modules. Red, increased expression; green, decreased expression. Middle of each panel: bar plots of the average expression of corresponding module eigengenes. Bottom of each panel: pie charts showing the abundance of lincRNAs and protein-coding genes and top Go terms of the later within each module.

Figure 5 Differential expression analysis of imprinted genes and qRT-PCR validation of RNA-seq.

(A): Heat map of DE imprinted genes. (B): qRT-PCR analysis of randomly selected imprinted genes, protein-coding genes and lincRNAs. Red line represents average expression of RNA-seq; Blue line represents average expression of qRT-PCR, and C value is the correlation between RNA-seq and qRT-PCR.

Supplementary Figure and Table Legends

Supplementary Figure S1 Identification and distribution of lincRNAs.

(A): Workflow for lincRNAs identification; (B): Distribution of LincRNA among different chromosome.

Supplementary Figure S2 Venn diagram of expressed genes during early embryonic development of IVF and PA.

(A): The Venn diagram shows the distribution of expressed genes of IVF or PA in different stages. (B): The Venn diagram shows the distribution of expressed genes of same stage between IVF and PA.

Supplementary Figure S3 Pearson correlation analysis between samples.

Supplementary Figure S4 Differential expression analysis between IVF and PA.

Histogram of DEGs in adjacent stages, IVF (A) and PA (B). (C): Heat map of differential expressed genes among morula and early blastocyst stage in IVF, and functional enrichment map was shown D; (E): Heat map of differential expressed genes among zygote and morula stage in PA, and functional enrichment map was shown F.

Supplementary Figure S5 Characteristics and differential expression analysis of lincRNAs.

(A): Transcript length distribution of lincRNAs and protein coding gene; (B): Distribution of exon length in lincRNAs and protein coding gene; (C): Distribution of exon number in lincRNAs and protein coding gene. (D): Comparison of average expression level between lincRNAs and protein-coding genes. Histogram of differentially expressed known and novel lincRNAs between adjacent stages of group IVF (E) and group PA (F).

Supplementary Figure S6 Co-expressed network visualization of hub lincRNAs and protein-coding genes in module green (A) and module greenyellow (B).

Supplementary Table S1 Primers for qRT-PCR

Supplementary Table S2 Reaction system of real-time quantitative PCR

Supplementary Table S3 Information of differential expression genes in this study

Supplementary Table S4 Gene ontology and pathway analysis of DEGs in a same stage

Supplementary Table S5-7 Information of putative lincRNAs (known/ Novel) and putative DE lincRNAs in this study

Supplementary Table S8 Information of lincRNAs neighboring protein-coding genes

Supplementary Table S9 Gene ontology and pathway analysis of protein-coding neighbors

Supplementary Table S10 Gene ontology and pathway analysis of protein-coding genes in six modules
