# Supplementary figures and images for: Transcriptome Analysis of In Vitro Fertilization and Parthenogenesis Activation during Early Embryonic Development in Pigs
Source: Genes (Basel). 2021 Sep 22;12(10):1461. doi: 10.3390/genes12101461 (PMC8535918; doi:10.3390/genes12101461)

A

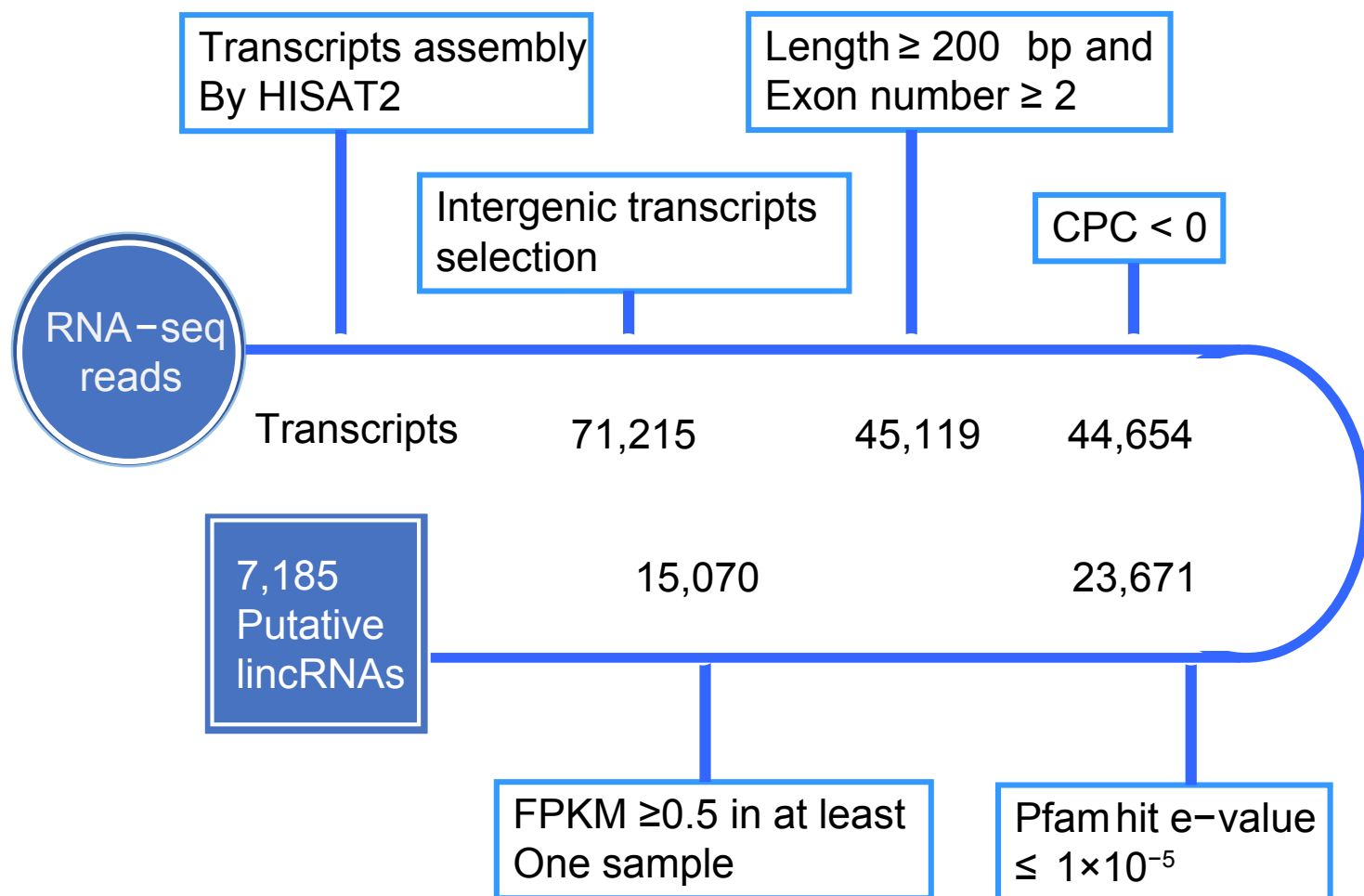

B

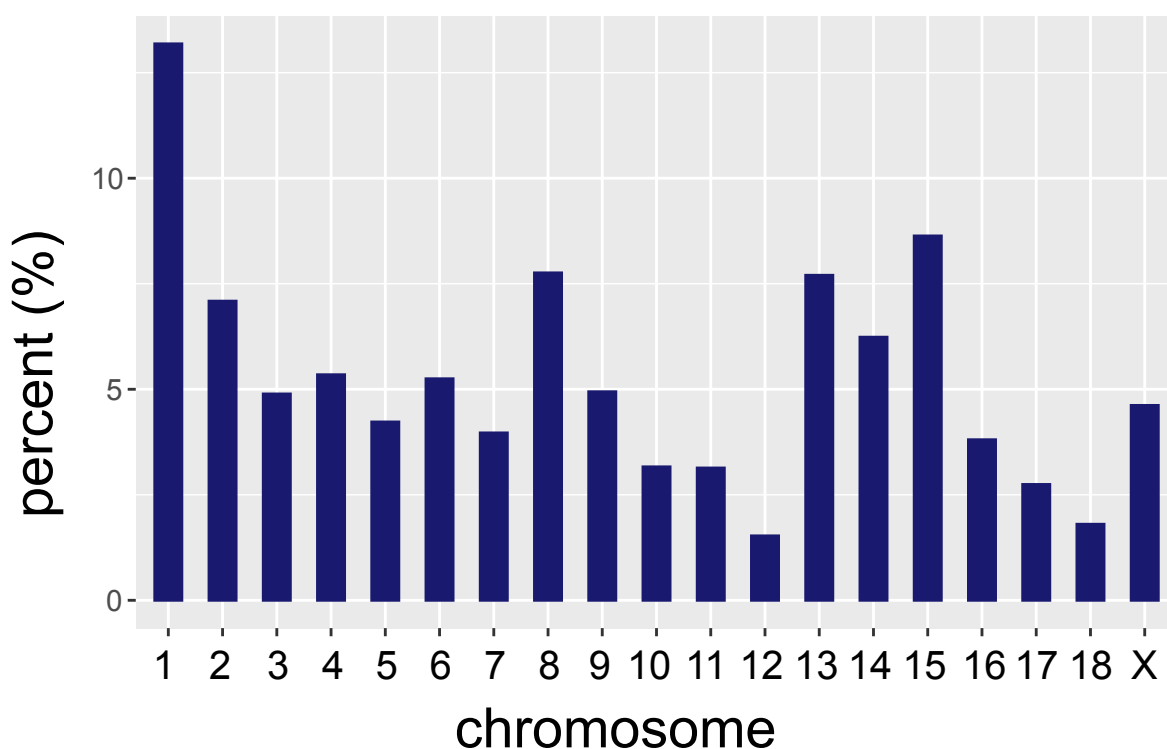

Supplement: Supplementary file 1 [file genes-12-01461-s001.zip › supplementary fig S1 pipeline.pdf]

Pearson correlation analysis between samples

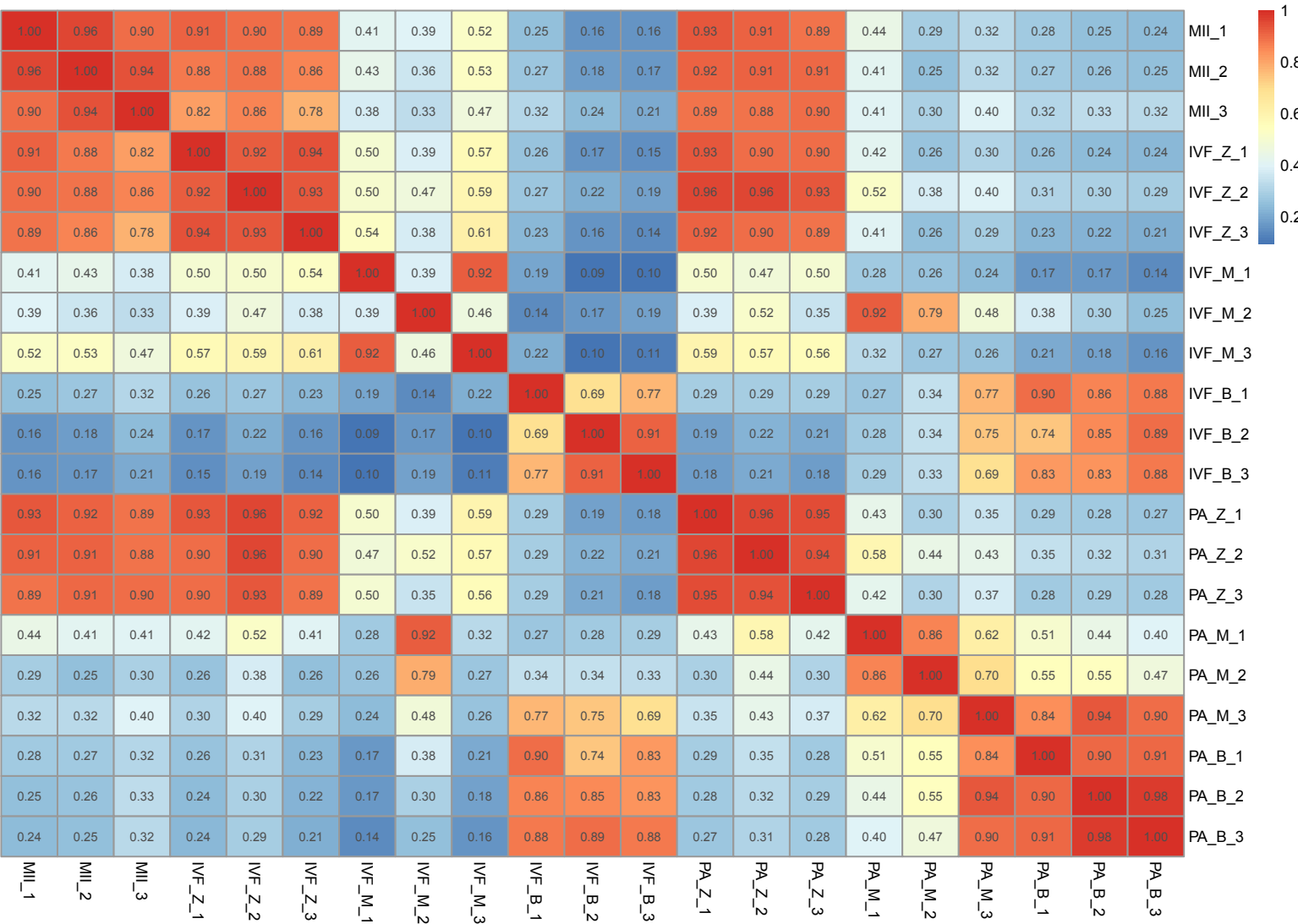

Supplement: Supplementary file 1 [file genes-12-01461-s001.zip › supplementary fig S3 correlation.pdf]

A

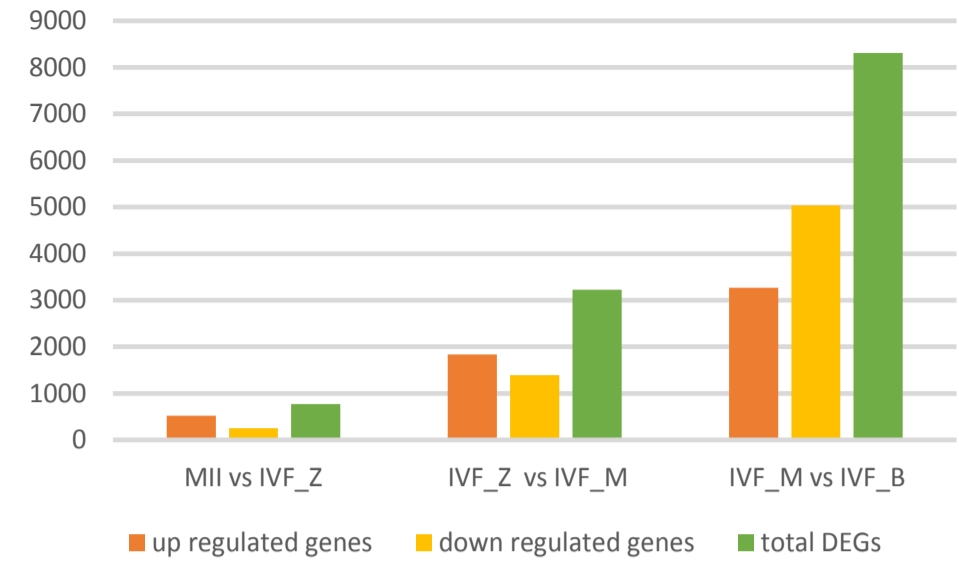

B

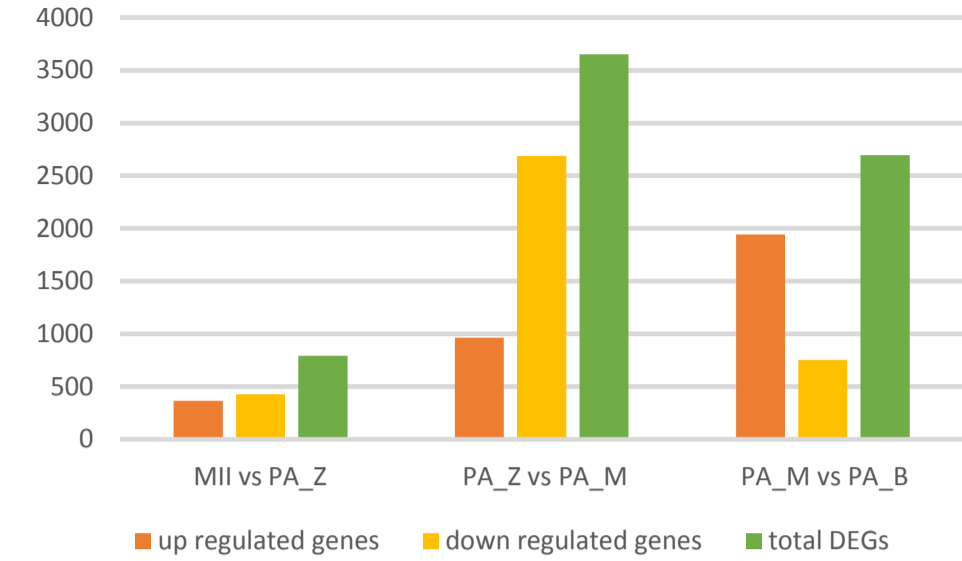

C

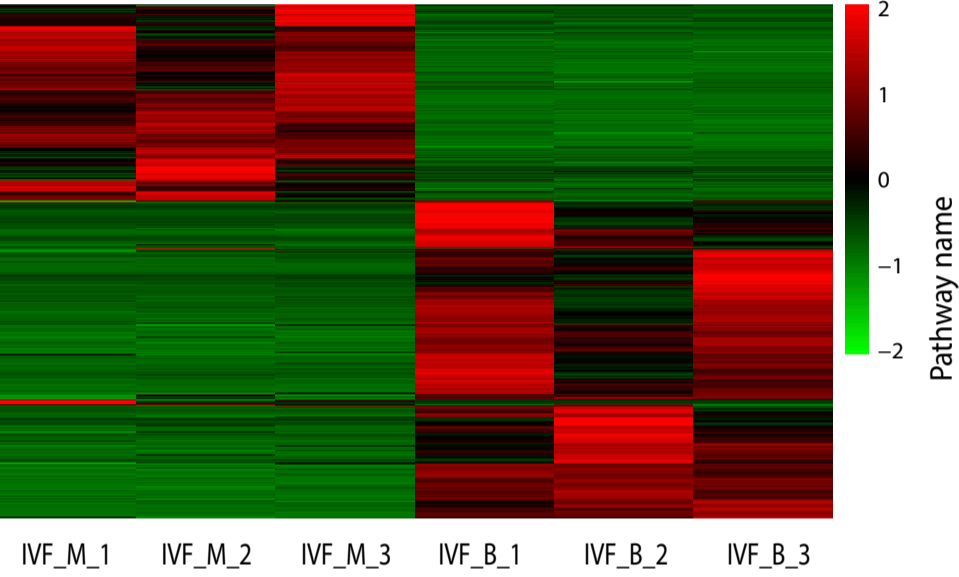

D

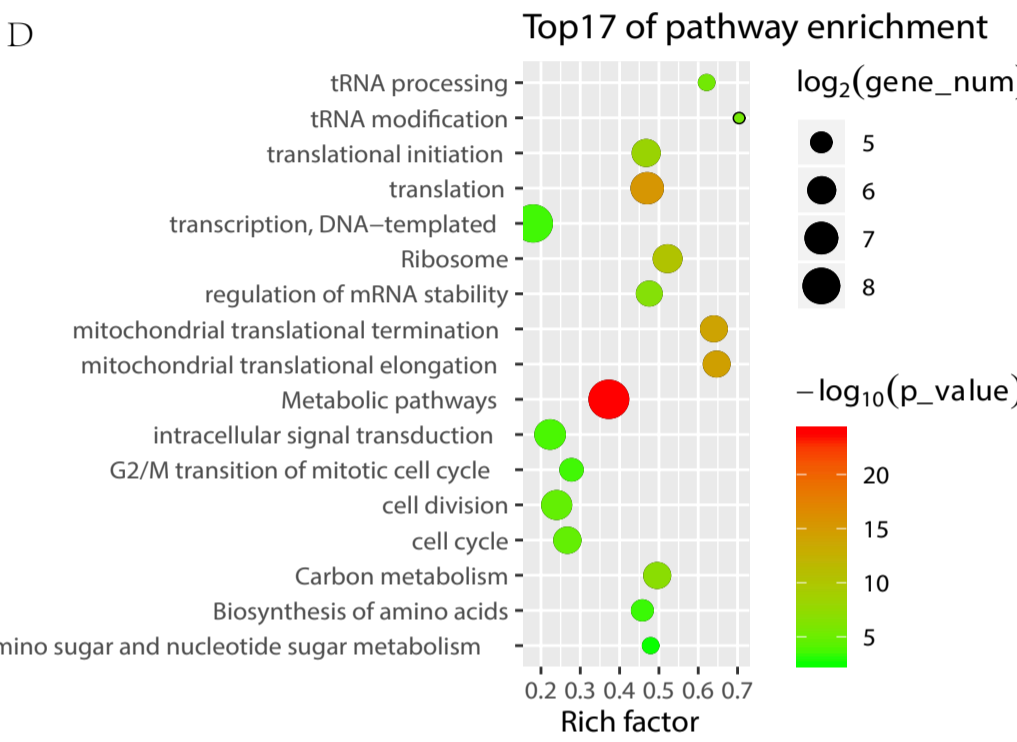

E

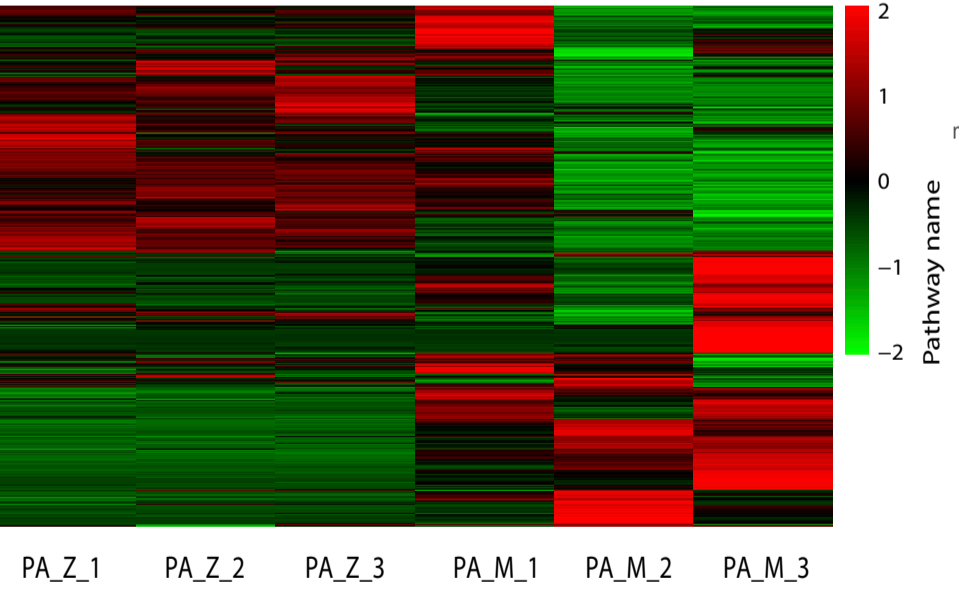

F

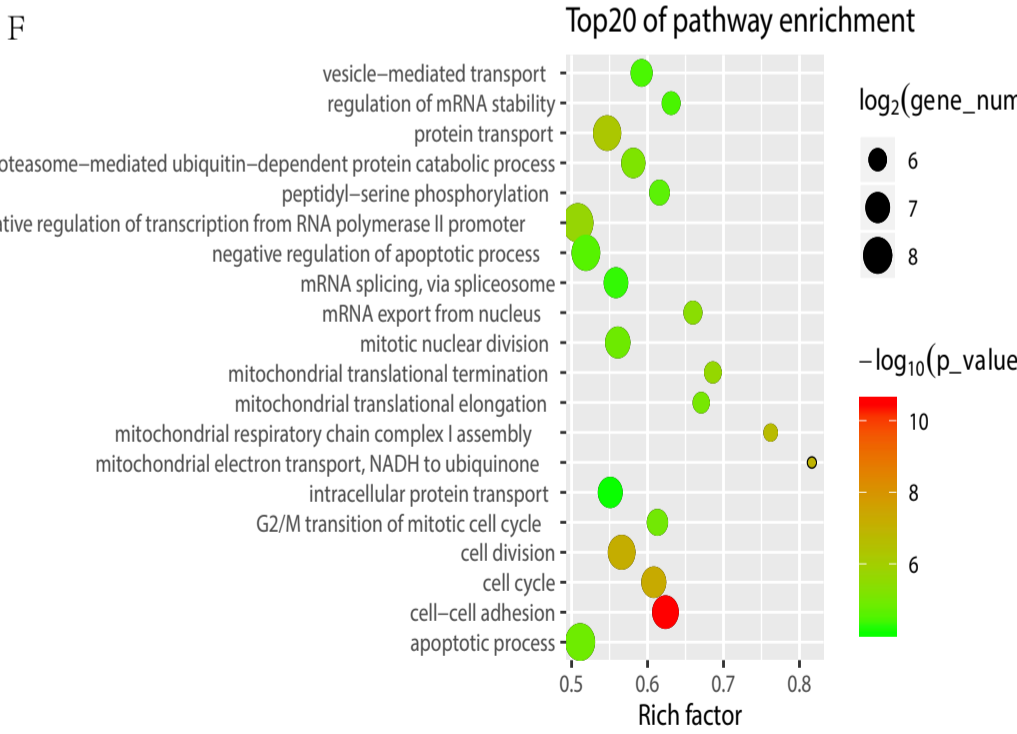

Supplement: Supplementary file 1 [file genes-12-01461-s001.zip › supplementary fig S4 DEG_IVF_PA.pdf]

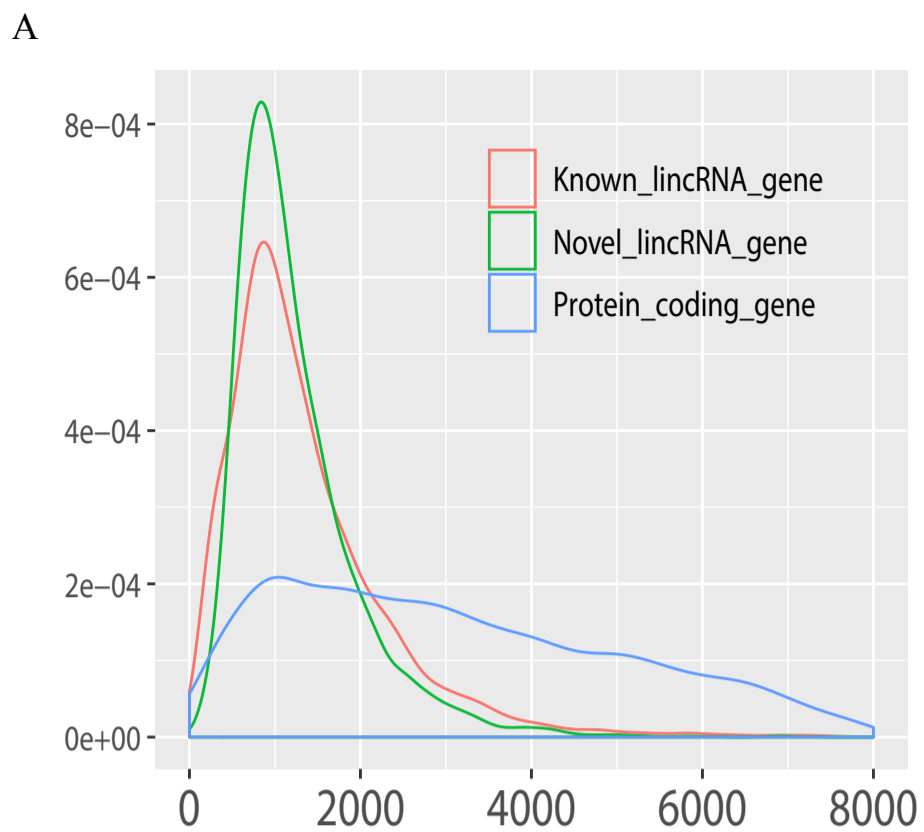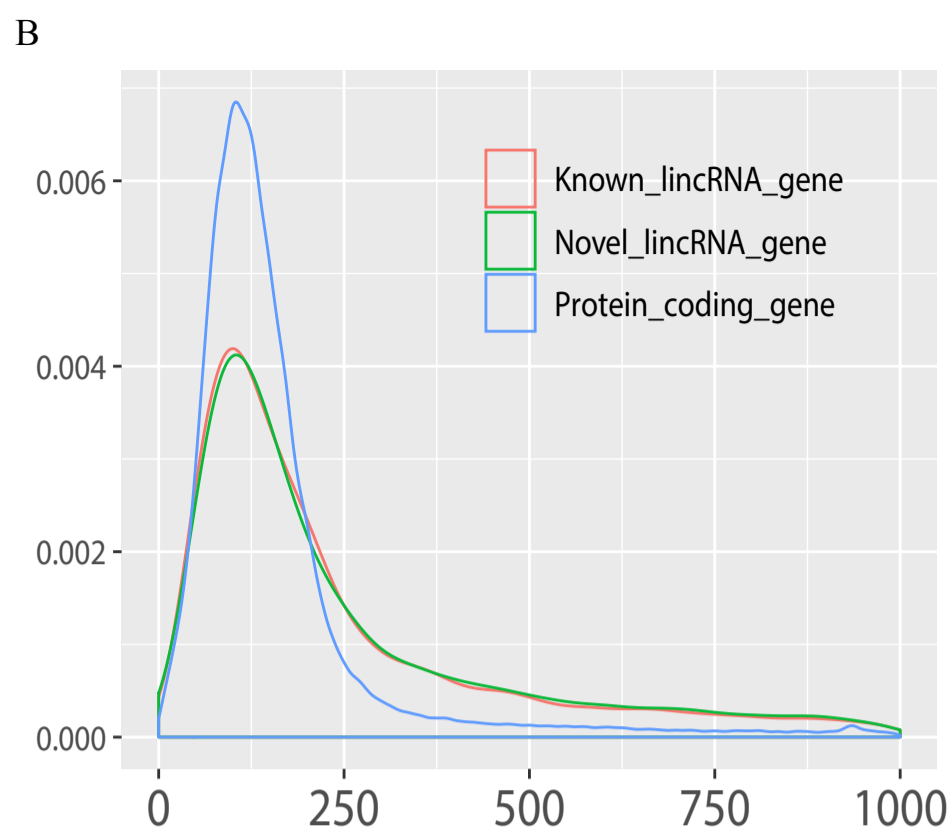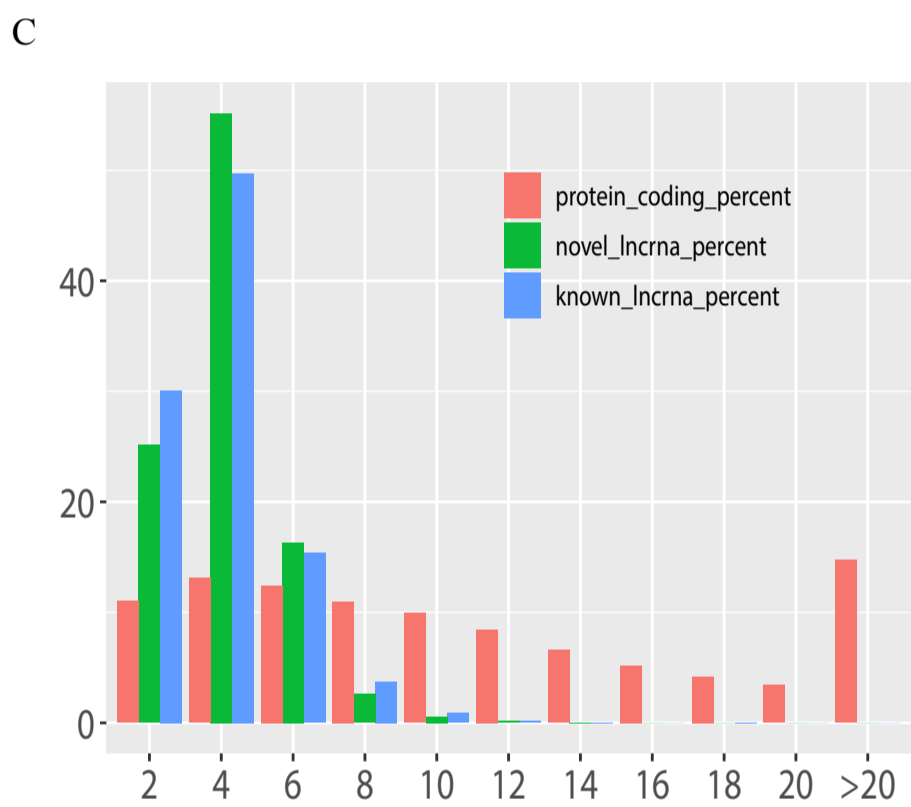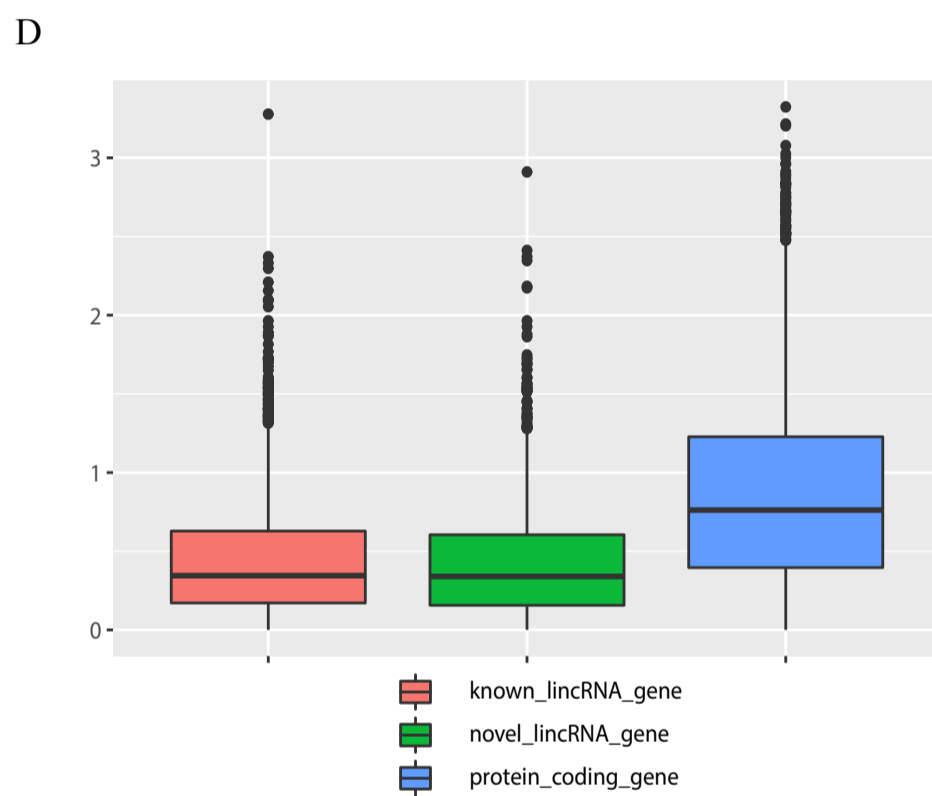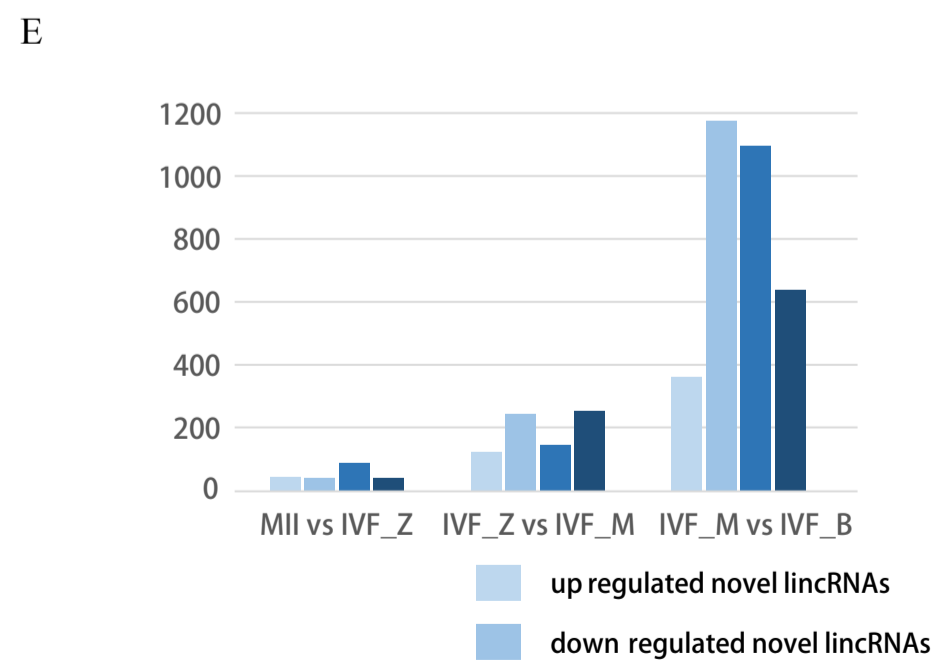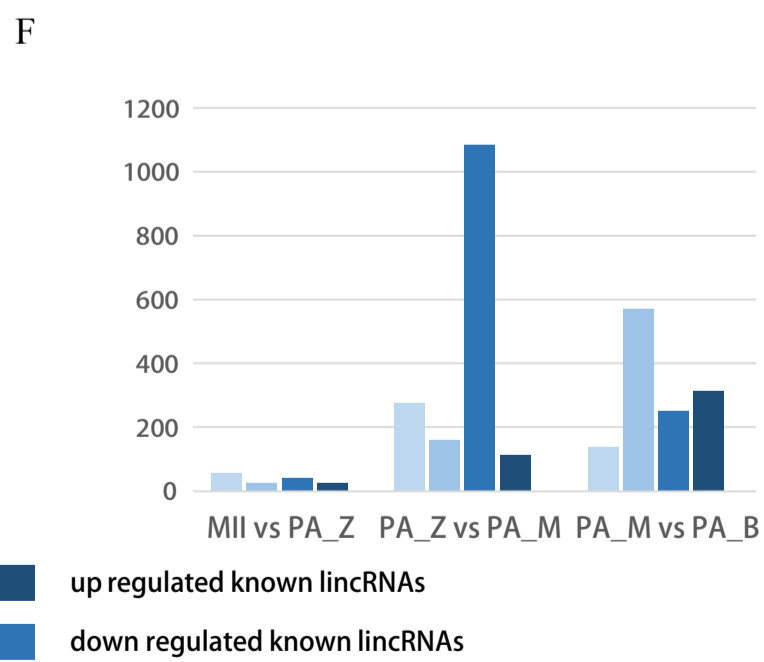

Supplement: Supplementary file 1 [file genes-12-01461-s001.zip › supplementary fig S5 A_F linc_character.pdf]

A

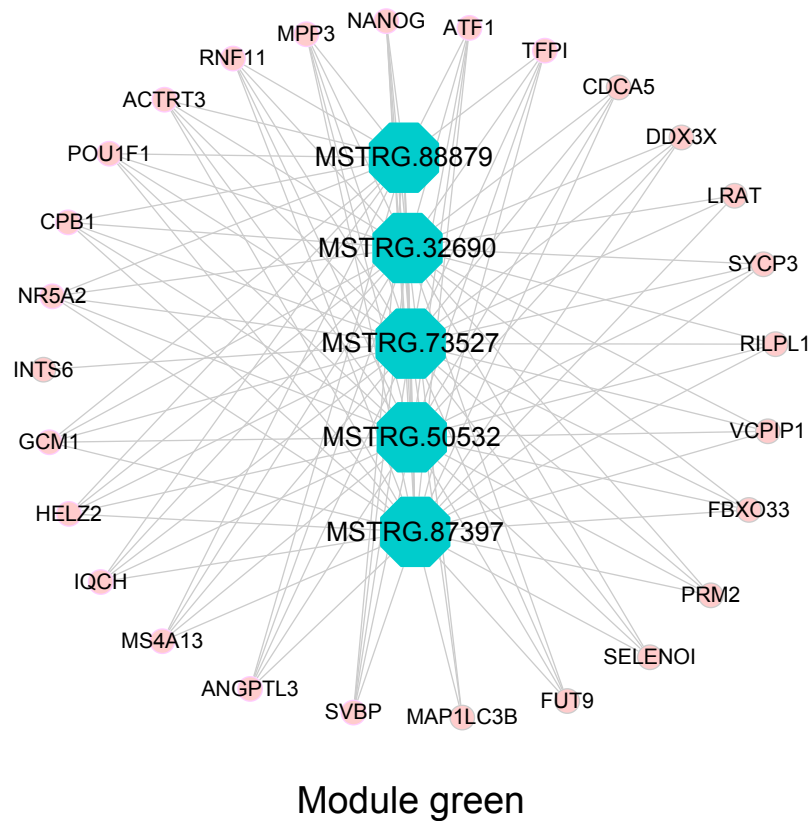

B

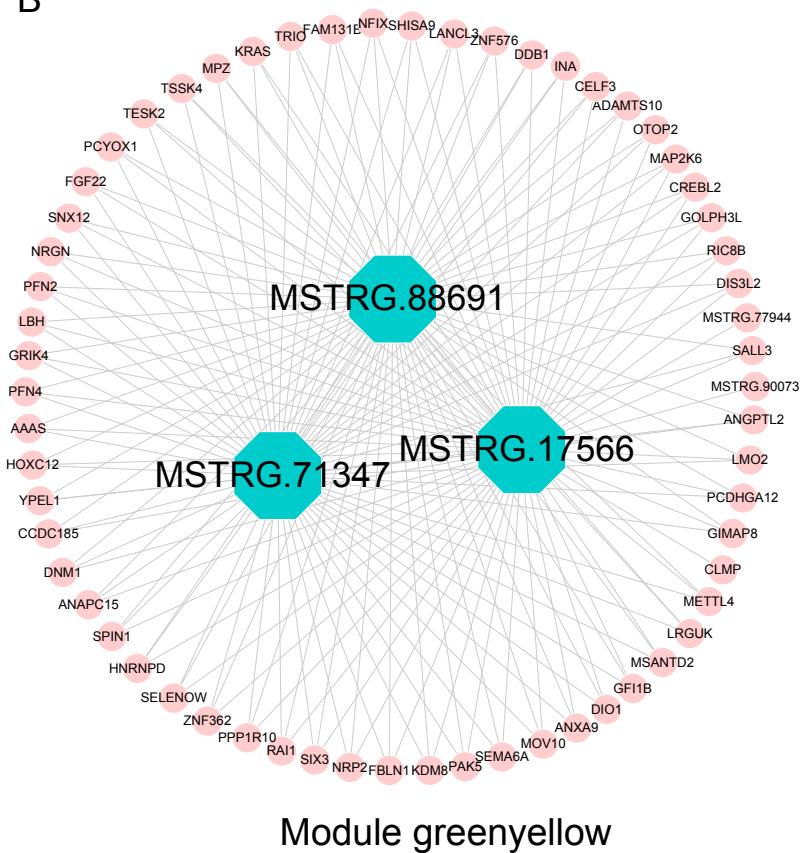

Supplement: Supplementary file 1 [file genes-12-01461-s001.zip › supplementary fig S6 WGCNA.pdf]
